# Metabolically unhealthy normal weight is associated with subclinical carotid atherosclerosis: cross-sectional and longitudinal evidence from a Chinese health examination cohort

**DOI:** 10.3389/fendo.2026.1925899

**Published:** 2026-09-16

**Authors:** Sai Zhang, Hui An, Hongmei Liu, Hao Wu, Zhifeng Tian

**Affiliations:** 1 Health Management & Physical Examination Center, Xiangyang Central Hospital, Affiliated Hospital of Hubei University of Arts and Science, Xiangyang, Hubei, China; 2 School of Public Health, Tongji Medical College, Huazhong University of Science and Technology, Wuhan, Hubei, China; 3 Department of Obstetrics and Gynecology, Xiangyang Central Hospital, Affiliated Hospital of Hubei University of Arts and Science, Xiangyang, Hubei, China

**Keywords:** cardiovascular risk, carotid intima-media thickness, metabolically unhealthy normal weight (MUHNW), obesity phenotypes, subclinical carotid atherosclerosis

## Abstract

**Background and aims:**

Whether metabolically unhealthy normal weight (MUNW) is associated with subclinical carotid atherosclerosis (SCAS) and how its risk compares with that of metabolically unhealthy overweight/obesity (MUO) remain uncertain, particularly in longitudinal analyses and across age groups.

**Methods:**

A cross-sectional sample (n = 26,202) and a longitudinal subcohort (n = 4,938) were derived from a health examination database; all participants in the longitudinal subcohort were free of SCAS at baseline. Participants were classified into four metabolic-obesity phenotypes based on body mass index and metabolic health status: metabolically healthy normal weight (MHNW), metabolically healthy overweight/obesity (MHO), metabolically unhealthy normal weight (MUNW), and metabolically unhealthy overweight/obesity (MUO). SCAS was defined as carotid intima-media thickness ≥1.0 mm and/or carotid plaque.

**Results:**

Compared with metabolically healthy normal weight (MHNW), MUNW was associated with increased odds of prevalent SCAS (OR 1.55, 95% CI 1.30–1.84) and increased risk of incident SCAS (HR 1.69, 95% CI 1.12–2.56). Compared with MUO, MUNW showed a numerically higher risk estimate for prevalent SCAS, although the difference was not statistically significant (OR 1.11, 95% CI 0.93–1.32, *P* = 0.247); a similar pattern was observed for incident SCAS (HR 1.25, 95% CI 0.83–1.91, *P* = 0.290). Metabolically healthy overweight/obesity (MHO) was associated with prevalent but not incident SCAS (OR 1.10, 95% CI 1.02–1.20; HR 1.14, 95% CI 0.94–1.38). A significant phenotype-by-age interaction was observed (*P* = 0.029); among adults aged <50 years, MUNW was associated with more than twofold higher odds of prevalent SCAS (OR 2.33, 95% CI 1.39–3.76). Findings were consistent in sensitivity analyses excluding covariates overlapping with phenotype definitions and in plaque-restricted analyses.

**Conclusions:**

MUNW was associated with increased odds of prevalent SCAS and a higher hazard of incident SCAS after multivariable adjustment, with numerically higher risk estimates than MUO, although direct comparisons did not reach statistical significance.

## 1 Introduction

Cardiovascular disease remains the leading cause of death in China, and metabolic syndrome affects a substantial proportion of the adult population (1, 2). The relative cardiovascular risk associated with excess adiposity and metabolic dysfunction remains uncertain, particularly when the two are dissociated: individuals with overweight/obesity but no metabolic abnormalities (metabolically healthy overweight/obesity, MHO), and individuals with normal weight but clear metabolic dysfunction (metabolically unhealthy normal weight, MUNW). The concept of a metabolically unhealthy normal-weight phenotype was first proposed over four decades ago (3), and cross-classifying weight status and metabolic health status now yields four metabolic-obesity phenotypes—metabolically healthy normal weight (MHNW), metabolically healthy overweight/obesity (MHO), metabolically unhealthy normal weight (MUNW), and metabolically unhealthy overweight/obesity (MUO)—a framework increasingly used to refine cardiovascular risk stratification beyond body mass index (BMI) alone (4–6). Because metabolic dysfunction and excess adiposity are each independently associated with cardiovascular risk, MUO is often assumed to confer the highest risk among the four phenotypes.

This assumption, however, has not been consistently supported by empirical evidence. Meta-analyses of prospective cohorts addressing related phenotype comparisons have themselves reached differing conclusions: one analysis of 41 cohorts reported increased mortality and cardiac event risk among metabolically unhealthy normal-weight individuals (7), while a separate systematic review found that even metabolically healthy overweight/obesity carries excess cardiovascular risk relative to normal weight (8), together suggesting that adiposity status alone does not reliably stratify risk. At the level of individual cohort studies, the picture is similarly mixed: several Chinese occupational studies using metabolically unhealthy non-obesity definitions have reported risk estimates that were of a similar magnitude to or numerically higher than those observed for MUO (9, 10), while other community-based studies have reported a graded increase in risk from MHO to MUNW to MUO, consistent with the conventional assumption (11). A national cross-sectional study using two independent obesity definitions—BMI and body fat mass percentage—similarly found that MUO conferred greater cardiovascular risk than metabolically unhealthy non-obesity using either definition (12), reinforcing the view that adiposity may add risk beyond metabolic dysfunction alone in some populations. This inconsistency has not been resolved, and the studies reporting opposing patterns differ in population (occupational versus community-based), age structure, and the specific vascular outcome examined—any of which could plausibly explain the divergence. A related and equally unsettled question concerns whether MHO represents a durably low-risk state or a transitional phenotype that has not yet progressed to overt metabolic dysfunction (8, 11), a distinction that cross-sectional studies alone cannot resolve.

Most prior studies addressing the relative risk of MUNW and MUO have relied on carotid plaque alone as the outcome, or on cross-sectional designs only. Carotid intima-media thickness has been established as an independent predictor of myocardial infarction and stroke in large prospective cohorts (13, 14). Subclinical carotid atherosclerosis (SCAS)—a composite marker incorporating both carotid intima-media thickening and plaque formation, as defined by international consensus (15)—captures a broader spectrum of carotid vascular abnormalities than plaque alone and may therefore be informative for understanding how metabolic-obesity phenotypes relate to early atherosclerotic disease. Importantly, few studies have simultaneously examined these associations using both cross-sectional and longitudinal designs within the same population, while also evaluating whether age modifies the phenotype-risk relationship. This is a meaningful gap because age may modify the clinical expression of metabolic dysfunction: if the association with MUNW is evident only at older ages, it may partly reflect the accumulation of age-related cardiometabolic risk factors; if it is also evident among younger adults, it would suggest that metabolic dysfunction may identify individuals with early vascular abnormalities despite apparently favorable BMI and age profiles.

Health examination populations offer a particularly relevant setting in which to address these questions. Unlike occupational or community cohorts selected for specific demographic or employment characteristics, health examination populations provide a real-world setting in which individuals undergo routine assessment of cardiometabolic risk factors. This setting is therefore particularly relevant for evaluating whether normal-weight individuals with metabolic dysfunction may be overlooked by BMI-based risk stratification.

In this study, we leveraged a large health examination cohort with both cross-sectional and longitudinal data to examine the independent associations between metabolic-obesity phenotypes and SCAS. We aimed to: (1) characterize the associations among MHO, MUNW, and MUO, with particular attention to whether risk estimates for MUNW differed from those for MUO; (2) assess whether associations observed cross-sectionally were corroborated longitudinally; and (3) determine whether age modifies the association between metabolic-obesity phenotypes and SCAS, particularly among younger adults who may be considered low-risk on the basis of BMI alone.

## 2 Methods

### 2.1 Study design and setting

This study utilized data from a large, single-center health examination database at Xiangyang Central Hospital, China. Two analytic samples were derived from this database: a cross-sectional sample used to examine the association between metabolic-obesity phenotypes and prevalent SCAS, and a longitudinal subcohort with repeated examinations used to examine incident SCAS. The participant selection process for the cross-sectional and longitudinal analyses is illustrated in **Supplementary Figure S1**. This study was conducted in accordance with the Declaration of Helsinki and was approved by the Institutional Review Board of Xiangyang Central Hospital (approval number: Ethics No. 2026-IIT-059). Given the retrospective design and the use of anonymized routinely collected clinical data, the requirement for informed consent was waived by the Institutional Review Board.

### 2.2 Study population

*Cross-sectional sample.* Participants who underwent a routine health examination, including carotid ultrasonography and waist circumference measurement, were eligible for inclusion. Waist circumference was incorporated into the routine examination protocol starting in 2021; therefore, the cross-sectional analytic sample was restricted to examinations performed from 2021 onward. Individuals with missing data on key variables required for phenotype classification (body mass index [BMI], waist circumference, blood pressure, fasting blood glucose, triglycerides, and high-density lipoprotein cholesterol [HDL-C]) or for the outcome (carotid ultrasonography results) were excluded. Two participants with biologically implausible neutrophil-to-lymphocyte ratio (NLR) values (NLR > 20, attributable to apparent data entry errors in lymphocyte or neutrophil counts) were excluded after data quality review, yielding a final cross-sectional sample of 26,202 individuals.

*Longitudinal subcohort.* Among participants in the cross-sectional sample who were confirmed to be free of SCAS at baseline, 4,938 participants with at least one subsequent health examination, including repeat carotid ultrasonography, were included in the longitudinal subcohort. Thus, all 4,938 participants were free of SCAS at baseline. Follow-up time was calculated from the date of the baseline examination to the date of the examination at which incident SCAS was identified, or to the date of the last available examination for participants who did not develop SCAS during follow-up.

### 2.3 Definition of metabolic-obesity phenotypes

Participants were classified into four metabolic-obesity phenotypes based on the cross-classification of weight status and metabolic health status at baseline. Weight status was defined using BMI according to the World Health Organization criteria for Asian populations (16): normal weight (BMI <24 kg/m²) versus overweight/obesity (BMI ≥24 kg/m²).

Metabolic dysfunction was defined according to the National Cholesterol Education Program Adult Treatment Panel III (NCEP ATP III) framework (17), with Asian-specific waist circumference thresholds (≥90 cm for men and ≥85 cm for women) applied for abdominal obesity (18). The five metabolic components comprised elevated waist circumference (≥90 cm for men or ≥85 cm for women), elevated blood pressure (SBP ≥130 mmHg or DBP ≥85 mmHg, or a self-reported physician-diagnosed history of hypertension), elevated fasting glucose (≥5.6 mmol/L or a self-reported physician-diagnosed history of diabetes), elevated triglycerides (≥1.7 mmol/L), and reduced HDL-C (<1.0 mmol/L in men or <1.3 mmol/L in women). Participants meeting at least three of these five criteria were classified as metabolically unhealthy, whereas those meeting fewer than three criteria were classified as metabolically healthy. Current medication-use information was not available in the health examination database and was therefore not incorporated into phenotype classification.

Following Asian-specific BMI thresholds, BMI ≥24 kg/m² was classified as overweight/obesity; individuals in this category with and without metabolic dysfunction were referred to as metabolically unhealthy overweight/obesity (MUO) and metabolically healthy overweight/obesity (MHO), respectively. Individuals with BMI <24 kg/m² were classified as normal weight and further categorized as metabolically healthy normal weight (MHNW) or metabolically unhealthy normal weight (MUNW) according to metabolic health status.

### 2.4 Outcome definition

The primary outcome, SCAS was defined as the presence of carotid intima-media thickness (CIMT) ≥1.0 mm and/or carotid plaque (15), based on B-mode carotid ultrasonography performed by trained sonographers as part of the routine health examination. In the cross-sectional analysis, prevalent SCAS at baseline was the outcome. In the longitudinal analysis, incident SCAS was defined as the new occurrence of SCAS at any follow-up examination among participants who were free of SCAS at baseline. A secondary outcome, carotid plaque alone (without the intima-media thickening component), was used in a sensitivity analysis to assess the robustness of findings using a more specific marker of atherosclerotic disease.

Carotid ultrasonography was performed bilaterally, examining the common carotid artery (CCA), the extracranial segment of the internal carotid artery (ICA), and the external carotid artery (ECA). CIMT was measured bilaterally at the common carotid artery; the higher value between the left and right sides was used to determine SCAS status. Carotid plaque was defined as a focal structure encroaching into the arterial lumen with a measured intima-media thickness exceeding 1.5 mm, consistent with the Mannheim Carotid Intima-Media Thickness and Plaque Consensus (15). Plaque assessment additionally incorporated color Doppler flow imaging (CDFI) to evaluate flow characteristics at the lesion site. Intra- and inter-observer reproducibility for carotid ultrasound measurements were not formally assessed as part of this study.

### 2.5 Covariates

Potential covariates considered in multivariable analyses included age, sex, systolic blood pressure, LDL-C, eGFR, GGT, NLR, serum uric acid, hypertension, diabetes status, and fatty liver. The specific subset of covariates included in each model is detailed in the corresponding analysis below. Hypertension and diabetes status used as covariates in the longitudinal models were defined based on self-reported physician-diagnosed history. Current medication-use information was not available in the health examination database and was therefore not incorporated into phenotype classification or covariate definitions. eGFR was calculated using the CKD-EPI equation (19). Fatty liver was assessed by abdominal ultrasonography and defined according to the presence of hepatic steatosis reported in the ultrasound examination.

### 2.6 Statistical analysis

*Descriptive statistics.* Baseline characteristics were compared across the four metabolic-obesity phenotypes using one-way analysis of variance for approximately normally distributed continuous variables, the Kruskal-Wallis test for skewed continuous variables (triglycerides, GGT, NLR, and uric acid), and chi-square tests for categorical variables.

*Cross-sectional analysis.* Multivariable logistic regression was used to estimate odds ratios (ORs) and 95% confidence intervals (CIs) for the association between metabolic-obesity phenotype and prevalent SCAS, with MHNW as the reference category. Three sequential models were constructed: Model 1 adjusted for age and sex; Model 2 additionally adjusted for systolic blood pressure, LDL-C, and eGFR; Model 3 additionally adjusted for GGT, NLR, and uric acid.

*Longitudinal analysis.* Cox proportional hazards regression was used to estimate hazard ratios (HRs) and 95% confidence intervals (CIs) for the association between baseline metabolic-obesity phenotype and incident SCAS. Model 1 adjusted for age and sex; Model 2 additionally adjusted for LDL-C, eGFR, GGT, NLR, and uric acid; Model 3 additionally adjusted for hypertension and diabetes. To limit over-parameterization given the number of incident events, covariates were introduced in a hierarchical manner across the three models. The proportional hazards assumption was assessed using Schoenfeld residuals (20, 21); no significant violation was observed for the phenotype variable or the global model in any of the three models (**Supplementary Table S2**). Because systolic blood pressure in the cross-sectional models and hypertension and diabetes in the longitudinal models overlap conceptually with components used to define metabolic health, Model 3 estimates may have been conservative. Sensitivity analyses excluding these overlapping covariates were performed to assess the robustness of the findings.

*Direct comparison between MUNW and MUO.* As a secondary analysis, the reference category was changed to MUO to directly compare MUNW with MUO, in both the cross-sectional and longitudinal models, using the fully adjusted covariate set.

*Age-stratified analysis.* To assess whether the association between metabolic-obesity phenotype and SCAS was modified by age, participants were stratified into three prespecified age groups (<50, 50–60, and ≥60 years), and multivariable logistic regression was performed within each stratum, adjusting for sex, LDL-C, eGFR, GGT, NLR, and uric acid. A multiplicative interaction term between phenotype and age group was included in the overall model to formally test for effect modification.

*Sensitivity analyses.* Several sensitivity analyses were performed to assess the robustness of the primary findings: (1) restricting the outcome to carotid plaque alone, excluding the intima-media thickening component; (2) excluding longitudinal participants with less than one year of follow-up, unless an incident SCAS event occurred within this period; and (3) excluding potentially overlapping covariates from the fully adjusted models, specifically systolic blood pressure in the cross-sectional analysis and hypertension and diabetes in the longitudinal analysis.

*Visualization.* Kaplan-Meier cumulative incidence curves were constructed to illustrate the unadjusted cumulative incidence of SCAS by phenotype over the follow-up period. Because the MUNW subgroup contained relatively few participants at risk beyond 2 years of follow-up, Kaplan-Meier curves were truncated at 2 years to improve visual stability and interpretability, as relatively few MUNW participants remained under observation beyond this time point.

All statistical analyses were performed using R (version 4.2.1; R Foundation for Statistical Computing, Vienna, Austria) (22, 23). A two-sided *P* value < 0.05 was considered statistically significant.

## 3 Results

### 3.1 Study population and baseline characteristics

The cross-sectional analytic sample comprised 26,202 individuals (mean age, 50.3 years; 63.3% men) who underwent health examination at Xiangyang Central Hospital from 2021 onward. Based on the cross-classification of weight status and metabolic health status, participants were categorized into four phenotypes: MHNW (n = 11,625, 44.4%), MHO (n = 8,263, 31.5%), MUNW (n = 828, 3.2%), and MUO (n = 5,486, 20.9%). Baseline characteristics differed significantly across phenotypes (**Table 1**). Notably, the MUNW group was the oldest among the four phenotype groups (58.4 years, compared with 49.0 years in MHNW). Despite having a normal BMI (22.6 kg/m²), participants with MUNW exhibited substantial metabolic abnormalities, including elevated triglycerides and fasting blood glucose (6.69 mmol/L), as well as high prevalences of hypertension (39.0%) and diabetes (15.2%). Among participants without evidence of SCAS at baseline, 4,938 individuals with at least one follow-up examination were included in the longitudinal subcohort (MHNW, n = 2,338; MHO, n = 1,576; MUNW, n = 124; MUO, n = 900).

**Table 1:** Baseline characteristics of the cross-sectional sample by metabolic-obesity phenotype.

Characteristic	MHNW	MHO	MUNW	MUO	*P* value
n	11,625	8,263	828	5,486	
Age, years	48.99 (13.95)	50.08 (13.16)	58.40 (11.48)	52.23 (12.56)	<0.001
Male, n (%)	5,569 (47.9)	6,106 (73.9)	494 (59.7)	4,411 (80.4)	<0.001
Female, n (%)	6,056 (52.1)	2,157 (26.1)	334 (40.3)	1,075 (19.6)	
BMI, kg/m²	21.57 (1.71)	26.23 (1.97)	22.60 (1.21)	27.83 (2.65)	<0.001
Waist circumference, cm	75.60 (7.28)	87.68 (7.42)	82.74 (7.03)	94.47 (7.22)	<0.001
SBP, mmHg	121.11 (16.04)	128.23 (15.28)	137.23 (14.40)	137.91 (14.34)	<0.001
DBP, mmHg	73.16 (10.01)	78.62 (10.63)	80.64 (10.32)	84.72 (11.22)	<0.001
Triglycerides, mmol/L	1.04 [0.78–1.42]	1.29 [0.98–1.66]	2.20 [1.77–3.19]	2.21 [1.74–3.01]	<0.001
Total cholesterol, mmol/L	5.09 (0.97)	5.14 (0.96)	5.38 (1.16)	5.27 (1.09)	<0.001
LDL-C, mmol/L	2.74 (0.78)	2.92 (0.79)	2.95 (0.92)	2.94 (0.85)	<0.001
HDL-C, mmol/L	1.52 (0.35)	1.34 (0.28)	1.14 (0.28)	1.12 (0.26)	<0.001
Fasting blood glucose, mmol/L	5.19 (0.98)	5.29 (0.86)	6.69 (2.41)	6.29 (2.01)	<0.001
eGFR, mL/min/1.73m²	102.47 (13.72)	99.75 (13.56)	95.59 (13.19)	98.42 (14.02)	<0.001
GGT, U/L	17.00 [12.00–24.90]	24.00 [17.00–36.00]	26.00 [18.00–41.00]	34.00 [23.00–53.70]	<0.001
NLR	1.64 [1.27–2.11]	1.68 [1.34–2.12]	1.71 [1.32–2.22]	1.74 [1.38–2.19]	<0.001
Uric acid, μmol/L	307.00 [257.00–362.20]	362.00 [307.55–419.00]	346.00 [295.00–408.00]	385.05 [328.52–447.95]	<0.001
Hypertension, n (%)	1,184 (10.2)	1,574 (19.0)	323 (39.0)	3,388 (61.8)	<0.001
Diabetes, n (%)	209 (1.8)	97 (1.2)	126 (15.2)	451 (8.2)	<0.001
Fatty liver, n (%)	1,401 (12.1)	3,872 (46.9)	418 (50.5)	4,412 (80.4)	<0.001

Values are presented as mean (SD) for normally distributed continuous variables, median [IQR] for non-normally distributed continuous variables (triglycerides, GGT, NLR, and uric acid), and n (%) for categorical variables. *P* values are from one-way analysis of variance (normally distributed continuous variables), Kruskal-Wallis test (non-normally distributed continuous variables), or chi-square test (categorical variables) across the four phenotype groups. MHNW, metabolically healthy normal weight; MHO, metabolically healthy overweight/obesity; MUNW, metabolically unhealthy normal weight; MUO, metabolically unhealthy overweight/obesity; BMI, body mass index; SBP, systolic blood pressure; DBP, diastolic blood pressure; LDL-C, low-density lipoprotein cholesterol; HDL-C, high-density lipoprotein cholesterol; eGFR, estimated glomerular filtration rate; GGT, gamma-glutamyl transferase; NLR, neutrophil-to-lymphocyte ratio; IQR, interquartile range.

### 3.2 Prevalence and incidence of SCAS by phenotype

The prevalence of SCAS at baseline differed substantially across phenotypes, ranging from 22.9% in MHNW to 28.8% in MHO, 37.9% in MUO, and 50.5% in MUNW—the highest prevalence among the four groups.

During longitudinal follow-up, 633 incident SCAS events occurred among 4,938 participants (12.8% overall), with cumulative incidence proportions of 10.3% (240/2,338) in MHNW, 13.5% (213/1,576) in MHO, 21.0% (26/124) in MUNW, and 17.1% (154/900) in MUO (**Supplementary Table S5**).

### 3.3 Cross-sectional associations between metabolic-obesity phenotypes and SCAS

In multivariable logistic regression models using MHNW as the reference category, all three non-reference phenotypes were associated with significantly higher odds of prevalent SCAS across all three models (**Table 2**). In the fully adjusted model (Model 3), MUNW showed the largest point estimate for prevalent SCAS (OR 1.55, 95% CI: 1.30–1.84, *P* < 0.001), followed by MUO (OR 1.36, 95% CI: 1.24–1.49, *P* < 0.001) and MHO (OR 1.10, 95% CI: 1.02–1.20, *P* = 0.020). The ordering of point estimates—MUNW, followed by MUO and MHO—was consistent across all three sequential models.

**Table 2:** Cross-sectional associations between metabolic-obesity phenotype and prevalent subclinical carotid atherosclerosis (SCAS).

Phenotype	Model 1, OR (95% CI), *P*	Model 2, OR (95% CI), *P*	Model 3, OR (95% CI), *P*
MHNW	1 (reference)	1 (reference)	1 (reference)
MHO	1.22 (1.12–1.32), *P* < 0.001	1.12 (1.03–1.21), *P* = 0.007	1.10 (1.02–1.20), *P* = 0.020
MUO	1.69 (1.55–1.84), *P* < 0.001	1.41 (1.29–1.55), *P* < 0.001	1.36 (1.24–1.49), *P* < 0.001
MUNW	1.89 (1.60–2.23), *P* < 0.001	1.61 (1.35–1.91), *P* < 0.001	1.55 (1.30–1.84), *P* < 0.001

MHNW was the reference category. Model 1 adjusted for age and sex. Model 2 further adjusted for systolic blood pressure, LDL-C, and eGFR. Model 3 additionally adjusted for GGT, NLR, and uric acid. OR, odds ratio; CI, confidence interval; MHNW, metabolically healthy normal weight; MHO, metabolically healthy overweight/obesity; MUNW, metabolically unhealthy normal weight; MUO, metabolically unhealthy overweight/obesity.

### 3.4 Longitudinal associations between metabolic-obesity phenotypes and incident SCAS

In the fully adjusted Cox model (Model 3), MUNW remained associated with a higher hazard of incident SCAS (HR 1.69, 95% CI: 1.12–2.56, *P* = 0.013), consistent with the cross-sectional findings (**Table 3**). MHO, however, was not significantly associated with incident SCAS (HR 1.14, 95% CI: 0.94–1.38, *P* = 0.196), in contrast to its significant cross-sectional association. The proportional hazards assumption was satisfied for all models, including the phenotype variable (**Supplementary Table S2**). The combined cross-sectional and longitudinal effect estimates are presented in **Figure 1**. Kaplan–Meier cumulative incidence curves demonstrated significant differences in the unadjusted cumulative incidence of SCAS among the four metabolic-obesity phenotypes (log-rank P < 0.001; **Supplementary Figure S2**). The median follow-up duration was 1.78 years (IQR, 1.02–2.12 years), and 633 incident SCAS events occurred among the 4,938 participants (12.8%) (**Supplementary Table S5**). Because the number of MUNW participants remaining at risk declined substantially during follow-up, with only 36 of 124 remaining at risk at 2 years, the precision of longer-term estimates for this subgroup was limited.

**Table 3:** Longitudinal associations between baseline metabolic-obesity phenotype and incident subclinical carotid atherosclerosis (SCAS).

Phenotype	Model 1, HR (95% CI), *P*	Model 2, HR (95% CI), *P*	Model 3, HR (95% CI), *P*
MHNW	1 (reference)	1 (reference)	1 (reference)
MHO	1.16 (0.96–1.40), *P* = 0.130	1.14 (0.94–1.38), *P* = 0.199	1.14 (0.94–1.38), *P* = 0.196
MUO	1.39 (1.13–1.72), *P* = 0.002	1.38 (1.11–1.72), *P* = 0.004	1.36 (1.08–1.71), *P* = 0.010
MUNW	1.69 (1.13–2.53), *P* = 0.012	1.73 (1.15–2.61), *P* = 0.009	1.69 (1.12–2.56), *P* = 0.013

Reference category: MHNW. Model 1 adjusted for age and sex. Model 2 additionally adjusted for LDL-C, eGFR, GGT, NLR, and uric acid. Model 3 additionally adjusted for hypertension and diabetes on the basis of Model 2. HR, hazard ratio; CI, confidence interval; MHNW, metabolically healthy normal weight; MHO, metabolically healthy overweight/obesity; MUNW, metabolically unhealthy normal weight; MUO, metabolically unhealthy overweight/obesity.

**Figure 1:**
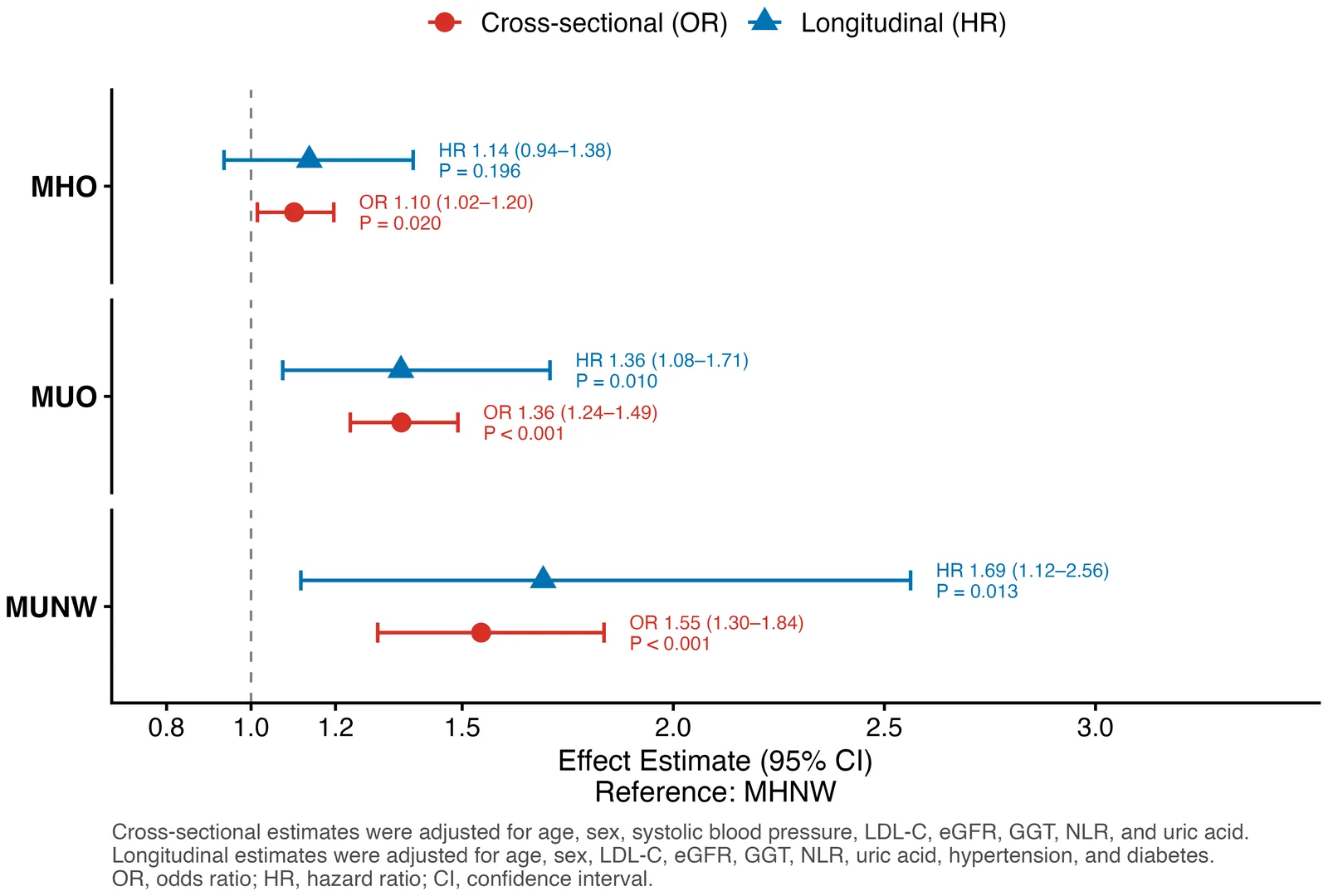
Forest plot of cross-sectional and longitudinal associations between metabolic-obesity phenotypes and subclinical carotid atherosclerosis (SCAS). Odds ratios (ORs) and hazard ratios (HRs) are shown for the cross-sectional and longitudinal analyses, respectively, using metabolically healthy normal weight (MHNW) as the reference group, based on the final multivariable-adjusted models (Model 3). Error bars represent 95% confidence intervals. Cross-sectional models were adjusted for age, sex, systolic blood pressure, LDL-C, eGFR, GGT, neutrophil-to-lymphocyte ratio (NLR), and uric acid. Longitudinal models were adjusted for age, sex, LDL-C, eGFR, GGT, NLR, uric acid, hypertension, and diabetes. OR, odds ratio; HR, hazard ratio; CI, confidence interval; MHNW, metabolically healthy normal weight; MHO, metabolically healthy overweight/obesity; MUNW, metabolically unhealthy normal weight; MUO, metabolically unhealthy overweight/obesity.

### 3.5 Direct comparison between MUNW and MUO

When the reference category was changed to MUO, MUNW showed a numerically higher risk estimate in both the cross-sectional (OR 1.11, 95% CI: 0.93–1.32, *P* = 0.247) and longitudinal analyses (HR 1.25, 95% CI: 0.83–1.91, *P* = 0.290), although neither comparison reached statistical significance.

### 3.6 Age-stratified analysis

The association between metabolic-obesity phenotype and SCAS differed significantly across age groups (phenotype × age-group interaction, *P* = 0.029). As shown in **Figure 2** and detailed in **Supplementary Table S4**, MUNW was associated with higher odds of SCAS in each age group, with the highest point estimate observed among participants aged <50 years (OR 2.33, 95% CI: 1.39–3.76, *P* < 0.001), followed by those aged 50–60 years (OR 2.06, 95% CI: 1.60–2.65, *P* < 0.001) and ≥60 years (OR 1.76, 95% CI: 1.39–2.26, *P* < 0.001). However, phenotype-specific pairwise interaction contrasts were not performed.

**Figure 2:**
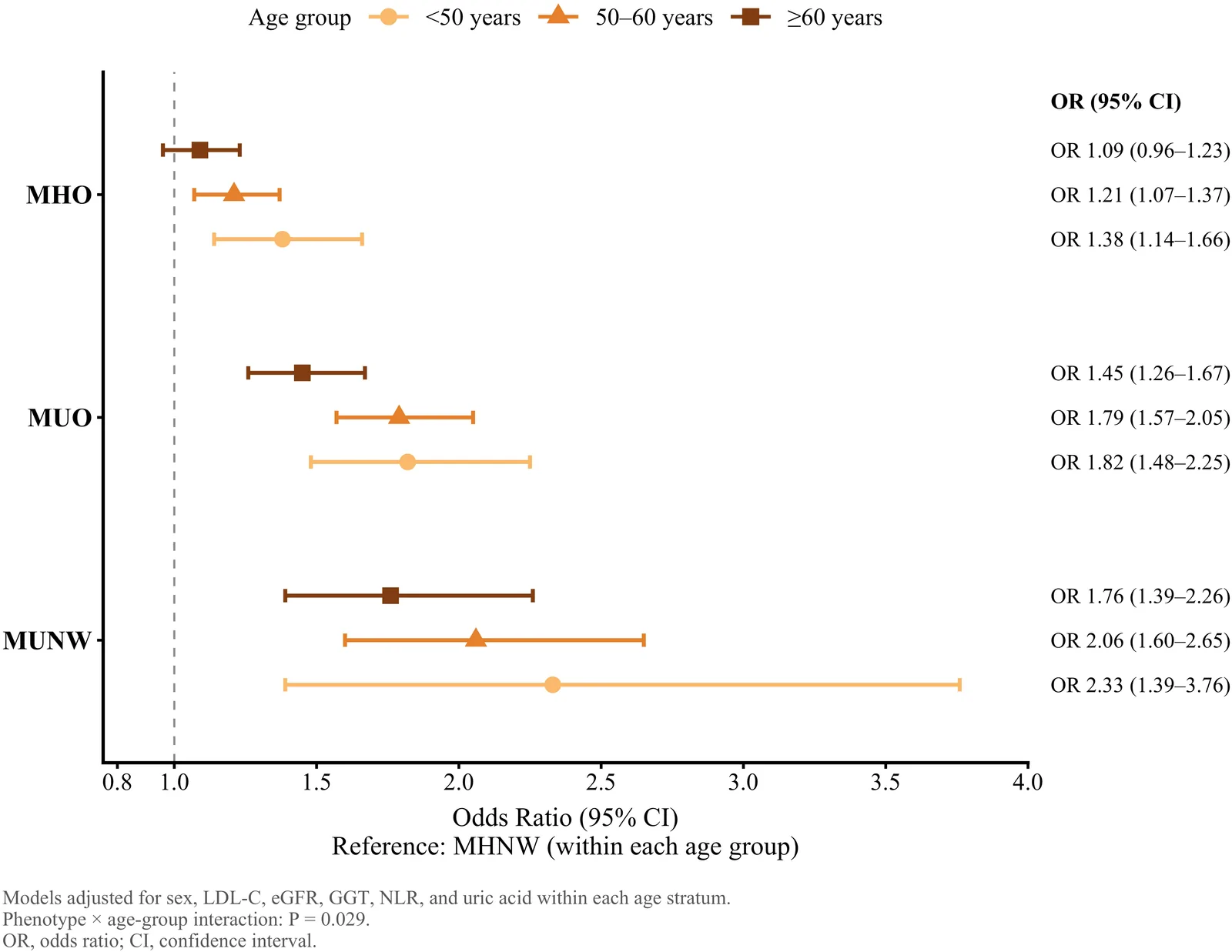
Forest plot of age-stratified associations between metabolic-obesity phenotypes and prevalent subclinical carotid atherosclerosis. Odds ratios (ORs) and 95% confidence intervals (CIs) are shown for each metabolic-obesity phenotype relative to metabolically healthy normal weight (MHNW) within the corresponding age group (<50, 50–60, and ≥60 years). Estimates were derived from multivariable logistic regression models adjusted for sex, LDL-C, estimated glomerular filtration rate (eGFR), gamma-glutamyl transferase (GGT), neutrophil-to-lymphocyte ratio (NLR), and uric acid within each age stratum. A significant phenotype-by-age interaction was observed (P for interaction = 0.029). OR, odds ratio; CI, confidence interval; MHNW, metabolically healthy normal weight; MHO, metabolically healthy overweight/obesity; MUNW, metabolically unhealthy normal weight; MUO, metabolically unhealthy overweight/obesity.

### 3.7 Sensitivity analyses

First, restricting the outcome to carotid plaque alone yielded results consistent with the primary analysis, with MUNW again showing the largest point estimate among the three phenotypes (OR 1.72, 95% CI: 1.45–2.04, *P* < 0.001), followed by MUO (OR 1.54, 95% CI: 1.41–1.69, *P* < 0.001) and MHO (OR 1.13, 95% CI: 1.04–1.23, *P* = 0.003) (**Supplementary Table S1**). Second, excluding longitudinal participants with less than one year of follow-up did not materially change the results (MUNW: HR 1.72, 95% CI: 1.14–2.59, *P* = 0.010; MUO: HR 1.39, 95% CI: 1.11–1.73, *P* = 0.004) (**Supplementary Table S3**). Third, to address the possibility that Model 3 was affected by adjustment for covariates overlapping with the phenotype definition, models were re-fitted excluding systolic blood pressure (cross-sectional) or hypertension and diabetes (longitudinal). Point estimates for MUNW were numerically higher in the sensitivity models than in the primary fully adjusted models (cross-sectional: OR 1.75, 95% CI: 1.47–2.07 vs. OR 1.55, 95% CI: 1.30–1.84; longitudinal: HR 1.73, 95% CI: 1.15–2.61 vs. HR 1.69, 95% CI: 1.12–2.56), suggesting that inclusion of these overlapping covariates may have attenuated the estimated associations. A similar pattern was observed for MHO and MUO (**Supplementary Table S6**).

## 4 Discussion

In this large health examination cohort, MUNW was consistently associated with higher odds of prevalent SCAS and a higher hazard of incident SCAS, despite the absence of excess weight. The point estimates for MUNW were numerically higher than those for MUO in both analyses, although direct comparisons did not reach statistical significance (cross-sectional OR 1.11, *P* = 0.247; longitudinal HR 1.25, *P* = 0.290). This pattern was broadly consistent in the plaque-restricted sensitivity analysis and across age strata in the cross-sectional analysis. MHO, by contrast, was associated with elevated odds of prevalent SCAS but not with incident SCAS during follow-up. By integrating cross-sectional and longitudinal analyses within the same health examination cohort, our study provides complementary evidence on the relationship between metabolic-obesity phenotypes and early carotid atherosclerosis.

These findings extend a body of literature on cardiovascular risk across metabolic-obesity phenotypes, although results have not been uniform. A study among 3,467 steelworkers in North China reported comparable carotid plaque risk between metabolically unhealthy non-obesity (MUNO) and MUO (adjusted OR 1.83 vs. 1.81, relative to a metabolically healthy non-obese reference), both substantially exceeding the risk in MHO (9). A cohort of 8,645 Chinese male railway drivers showed a similar pattern, with a numerically higher risk in MUNO than MUO (OR 2.20 vs. 1.78) (10). Although these two studies classified individuals as non-obese versus obese rather than using a strictly normal-weight cutoff, their findings provide related but not directly equivalent evidence, and our study extends this pattern using a stricter Asian normal-weight criterion.

In contrast, a large community-based cohort in Hunan Province reported a graded increase in risk from MHO (OR 1.10) to MUNW (OR 1.27) to MUO (OR 1.54), consistent with the conventional assumption that adiposity confers additional risk beyond metabolic dysfunction alone (11). A national cross-sectional study using both BMI and body fat mass percentage similarly found greater cardiovascular risk among individuals with MUO than among those with metabolically unhealthy non-obesity (12). These contrasting findings suggest that the relative contribution of adiposity and metabolic dysfunction may vary according to population characteristics, adiposity definition, age structure, and vascular outcome. Our findings were directionally consistent with the former pattern and extend these observations to a real-world health examination population using both cross-sectional and longitudinal analyses of SCAS.

Several unmeasured mechanisms may potentially contribute to this pattern. One possibility is that BMI does not adequately capture visceral adiposity. Individuals with a normal BMI but excess visceral fat can exhibit a metabolic profile resembling that of obesity (24). This discordance may be particularly relevant in East Asian populations, where visceral fat may accumulate at lower BMI thresholds than in Western populations (25). Thus, some normal-weight Asian adults may carry excess visceral or ectopic fat and an associated atherogenic metabolic profile despite an unremarkable BMI. This possibility cannot be directly evaluated in the present study because measures of visceral adiposity and body composition were unavailable.

Age-related decline in skeletal muscle mass and quality may also potentially contribute. Reduced muscle mass can promote fat accumulation, while increased fat mass may compromise muscle quality through fat infiltration and insulin resistance, creating a bidirectional process that can intensify with age without substantial changes in BMI (26). A nationwide Chinese cohort linked sarcopenia to incident cardiovascular disease specifically among normal-weight middle-aged and older adults (27). Although speculative, this mechanism may be relevant to the MUNW phenotype. The MUNW group was substantially older than the MHNW group in our cohort (58.4 vs. 49.0 years), but because direct measures of skeletal muscle mass and body composition were unavailable, this proposed mechanism remains untested and warrants further investigation.

Notably, the association between MUNW and SCAS was evident across all age strata, including adults younger than 50 years (OR 2.33, 95% CI: 1.39–3.76). Although the overall phenotype × age-group interaction was significant (P = 0.029), phenotype-specific interaction contrasts were not performed. Therefore, the higher point estimate observed among younger adults should be interpreted as suggestive rather than definitive evidence of stronger MUNW-specific effects at younger ages. Nevertheless, the presence of an elevated association in younger adults is clinically relevant because these individuals may otherwise be considered at relatively low risk when assessed primarily on the basis of age and BMI.

The pattern observed for MHO offers an informative contrast. MHO was associated with elevated SCAS risk cross-sectionally (OR 1.10, *P* = 0.020) but not longitudinally (HR 1.14, *P* = 0.196). The divergent cross-sectional and longitudinal findings for MHO may reflect phenotype heterogeneity, limited follow-up duration, or insufficient event accumulation, rather than establishing a benign or transitional nature. Longer follow-up with repeated assessment of metabolic status would be required to determine whether MHO represents a stable phenotype or a state that changes over time.

From a clinical perspective, these findings suggest that BMI alone may be insufficient for identifying vascular risk among normal-weight individuals. Metabolic dysfunction in the absence of excess adiposity was associated with increased odds of prevalent SCAS and a higher hazard of incident SCAS, while direct comparisons with MUO did not demonstrate statistically significant differences. The findings therefore support consideration of metabolic risk factors in cardiovascular risk assessment even among individuals who do not meet BMI-based criteria for excess weight.

Several limitations warrant mention. First, direct comparisons between MUNW and MUO were not statistically significant; therefore, our findings should be interpreted as evidence that MUNW showed numerically higher risk estimates than MUO, rather than definitive evidence of a difference in risk between the two phenotypes. Second, this was a single-center study based on a health examination population, which may limit generalizability. Third, the MUNW subgroup was relatively small in the longitudinal cohort (n = 124, 26 incident events), yielding wide confidence intervals; the number of participants remaining at risk declined substantially over follow-up, with only 36 of 124 MUNW participants remaining at risk at 2 years, further limiting the precision of longer-term estimates for this phenotype. In addition, the overall follow-up duration was relatively short (median 1.78 years); Kaplan-Meier curves were truncated at 2 years given the limited number of participants remaining at risk beyond this point, and longer-term SCAS incidence could not be evaluated. Fourth, because systolic blood pressure in the cross-sectional models and hypertension and diabetes in the longitudinal models overlap conceptually with components used to define metabolic health, Model 3 estimates may be conservative. Sensitivity analyses excluding these overlapping covariates yielded numerically higher point estimates for MUNW (OR 1.75 vs. 1.55 and HR 1.73 vs. 1.69), with the direction and statistical significance of the associations unchanged. Fifth, because metabolic abnormalities were incorporated into phenotype classification, the markedly higher triglycerides, fasting glucose, and prevalences of hypertension and diabetes observed in the MUNW group (**Table 1**) should be interpreted descriptively rather than as independent findings. Sixth, waist circumference, which was required for phenotype classification, was available only from 2021 onward, constraining the analytic sample and follow-up duration. Seventh, phenotypes were classified at baseline only, and transitions in metabolic-obesity status during follow-up were not captured. Eighth, this study lacked direct measures of visceral fat and skeletal muscle mass, limiting our ability to test the proposed mechanistic pathways directly. Ninth, information on smoking status, alcohol use, and medication use was unavailable in the health examination database, precluding adjustment for antihypertensive, lipid-lowering, or glucose-lowering therapies. Tenth, intra- and inter-observer reproducibility for carotid ultrasound measurements was not formally assessed. Finally, as an observational study, residual confounding cannot be excluded.

In conclusion, MUNW was associated with increased odds of prevalent SCAS and a higher hazard of incident SCAS after multivariable adjustment, with numerically higher point estimates than MUO, although direct comparisons did not reach statistical significance.

## Data Availability

The data that support the findings of this study are not publicly available because they contain potentially identifiable health information and are subject to institutional and ethical restrictions. Data may be made available from the corresponding author upon reasonable request and with permission from the relevant institutional and ethics committees. Requests to access these datasets should be directed to ZT, Tianzhifeng66@126.com.

## References

[1] Liu J Qi J Yin P Liu Y You J Lin L . Cardiovascular disease mortality — China, 2019. China CDC Wkly. (2021) 3:323–6. doi: 10.46234/ccdcw2021.087 34594876 PMC8393177

[2] Yao F Bo Y Zhao L Li Y Ju L Fang H . Prevalence and influencing factors of metabolic syndrome among adults in China from 2015 to 2017. Nutrients. (2021) 13:4475. doi: 10.3390/nu13124475 34960027 PMC8705649

[3] Ruderman NB Schneider SH Berchtold P . The "metabolically-obese," normal-weight individual. Am J Clin Nutr. (1981) 34:1617–21. doi: 10.1093/ajcn/34.8.1617 7270486

[4] Karelis AD Brochu M Rabasa-Lhoret R . Can we identify metabolically healthy but obese individuals (MHO)? Diabetes Metab. (2004) 30:569–72. doi: 10.1016/S1262-3636(07)70156-8 15671927

[5] Stefan N Häring HU Hu FB Schulze MB . Metabolically healthy obesity: epidemiology, mechanisms, and clinical implications. Lancet Diabetes Endocrinol. (2013) 1:152–62. doi: 10.1016/S2213-8587(13)70062-7 24622321

[6] Wildman RP Muntner P Reynolds K McGinn AP Rajpathak S Wylie-Rosett J . The obese without cardiometabolic risk factor clustering and the normal weight with cardiometabolic risk factor clustering: prevalence and correlates of 2 phenotypes among the US population (NHANES 1999-2004). Arch Intern Med. (2008) 168:1617–24. doi: 10.1001/archinte.168.15.1617 18695075

[7] Putra ICS Kamarullah W Prameswari HS Pramudyo M Iqbal M Achmad C . Metabolically unhealthy phenotype in normal weight population and risk of mortality and major adverse cardiac events: a meta-analysis of 41 prospective cohort studies. Diabetes Metab Syndr. (2022) 16:102635. doi: 10.1016/j.dsx.2022.102635 36240685

[8] Opio J Croker E Odongo GS Attia J Wynne K McEvoy M . Metabolically healthy overweight/obesity are associated with increased risk of cardiovascular disease in adults, even in the absence of metabolic risk factors: a systematic review and meta-analysis of prospective cohort studies. Obes Rev. (2020) 21:e13127. doi: 10.1111/obr.13127 32869512

[9] Yu M Zhang S Wang L Wu J Li X Yuan J . Metabolically healthy obesity and carotid plaque among steelworkers in North China: the role of inflammation. Nutrients. (2022) 14:5123. doi: 10.3390/nu14235123 36501152 PMC9740549

[10] Pan J Wang Z Dong C Yang B Tang L Jia P . Association between obese phenotypes and risk of carotid artery plaque among Chinese male railway drivers. BMC Public Health. (2022) 22:1859. doi: 10.1186/s12889-022-14253-y 36199053 PMC9533504

[11] Huang Q Liu Z Wei M Feng J Huang Q Liu Y . Metabolically healthy obesity, transition from metabolic healthy to unhealthy status, and carotid atherosclerosis. Diabetes Metab Res Rev. (2024) 40:e3766. doi: 10.1002/dmrr.3766 38351639

[12] Liu JT Yao HY Yu SC Liu JJ Zhu GJ Han SM . Joint association of metabolic health and obesity with ten-year risk of cardiovascular disease among Chinese adults. BioMed Environ Sci. (2022) 35:13–21. doi: 10.3967/bes2022.003 35078558

[13] O'Leary DH Polak JF Kronmal RA Manolio TA Burke GL Wolfson SK Jr . Carotid-artery intima and media thickness as a risk factor for myocardial infarction and stroke in older adults. N Engl J Med. (1999) 340:14–22. doi: 10.1056/NEJM199901073400103 9878640

[14] Chambless LE Heiss G Folsom AR Rosamond W Szklo M Sharrett AR . Association of coronary heart disease incidence with carotid arterial wall thickness and major risk factors: the Atherosclerosis Risk in Communities (ARIC) Study, 1987-1993. Am J Epidemiol. (1997) 146:483–94. doi: 10.1093/oxfordjournals.aje.a009302 9290509

[15] Touboul PJ Hennerici MG Meairs S Adams H Amarenco P Bornstein N . Mannheim carotid intima-media thickness and plaque consensus (2004-2006-2011). Cerebrovasc Dis. (2012) 34:290–6. doi: 10.1159/000343145 23128470 PMC3760791

[16] WHO/IASO/IOTF . The Asia-Pacific Perspective: Redefining Obesity and its Treatment. Sydney: Health Communications Australia (2000).

[17] Expert Panel on Detection, Evaluation, and Treatment of High Blood Cholesterol in Adults . Executive summary of the third report of the National Cholesterol Education Program (NCEP) Expert Panel on Detection, Evaluation, and Treatment of High Blood Cholesterol in Adults (Adult Treatment Panel III). JAMA. (2001) 285:2486–97. doi: 10.1001/jama.285.19.2486 11368702

[18] Bao Y Lu J Wang C Yang M Li H Zhang X . Optimal waist circumference cutoffs for abdominal obesity in Chinese. Atherosclerosis. (2008) 201:378–84. doi: 10.1016/j.atherosclerosis.2008.03.001 18417137

[19] Levey AS Stevens LA Schmid CH Zhang YL Castro AF 3rd Feldman HI . A new equation to estimate glomerular filtration rate. Ann Intern Med. (2009) 150:604–12. doi: 10.7326/0003-4819-150-9-200905050-00006 19414839 PMC2763564

[20] Schoenfeld D . Partial residuals for the proportional hazards regression model. Biometrika. (1982) 69:239–41. doi: 10.1093/biomet/69.1.239

[21] Grambsch PM Therneau TM . Proportional hazards tests and diagnostics based on weighted residuals. Biometrika. (1994) 81:515–26. doi: 10.1093/biomet/81.3.515

[22] Therneau TM Grambsch PM . Modeling Survival Data: Extending the Cox Model. New York: Springer (2000).

[23] R Core Team . R: A Language and Environment for Statistical Computing. Vienna: R Foundation for Statistical Computing (2022).

[24] Lu YC Lin YC Yen AM Chan WP . Dual-energy X-ray absorptiometry-assessed adipose tissues in metabolically unhealthy normal weight Asians. Sci Rep. (2019) 9:17698. doi: 10.1038/s41598-019-53557-9 31776349 PMC6881341

[25] Lim S Ji L Tham KW Misra A Kadowaki T . Clinical obesity in Asian people: bridging the gap between adiposity and disease. Nat Rev Endocrinol. (2026) 22:615–24. doi: 10.1038/s41574-026-01239-8 42045672

[26] Yu M Ge L Fu C Zhao R . Unraveling the metabolic pathways between atherosclerosis and sarcopenia. Front Endocrinol. (2026) 16:1762825. doi: 10.3389/fendo.2025.1762825 41726101 PMC12920195

[27] Cheng H Jia Z Chen J Xie YJ Hernandez J Wang HHX . Relationship between sarcopenia and cardiovascular disease among middle-aged and older adults with normal weight in China: functional limitation plays a mediating role. Environ Health Prev Med. (2025) 30:46. doi: 10.1265/ehpm.24-00351 40467456 PMC12170099

