# Supplementary material for: Metabolically unhealthy normal weight is associated with subclinical carotid atherosclerosis: cross-sectional and longitudinal evidence from a Chinese health examination cohort
Source: Front Endocrinol (Lausanne). 2026 Sep 16;17:1925899. doi: 10.3389/fendo.2026.1925899 (PMC13623737; doi:10.3389/fendo.2026.1925899)
Supplement: Supplementary file 1 [file SupplementaryFile1.docx]

**Supplementary Material**

Metabolically Unhealthy Normal Weight Is Associated With Subclinical Carotid Atherosclerosis:

Cross-Sectional and Longitudinal Evidence From a Chinese Health Examination Cohort

Sai Zhang, Hui An, Hongmei Liu, Hao Wu, Zhifeng Tian

**Table S1.** Sensitivity analysis restricting the outcome to carotid plaque alone (fully adjusted model).

| **Phenotype** | **OR (95% CI)** | ***P* value** |
| --- | --- | --- |
| MHNW | 1 (reference) | – |
| MHO | 1.13 (1.04–1.23) | 0.003 |
| MUO | 1.54 (1.41–1.69) | <0.001 |
| MUNW | 1.72 (1.45–2.04) | <0.001 |

Reference category: MHNW. Fully adjusted for age, sex, systolic blood pressure, LDL-C, eGFR, GGT, NLR, and uric acid. OR, odds ratio; CI, confidence interval; MHNW, metabolically healthy normal weight; MHO, metabolically healthy overweight/obesity; MUNW, metabolically unhealthy normal weight; MUO, metabolically unhealthy overweight/obesity.

**Table S2.** Schoenfeld residual test of the proportional hazards assumption (fully adjusted Cox model, Model 3).

| **Variable** | **Chi-square** | **df** | ***P* value** |
| --- | --- | --- | --- |
| phenotype | 0.9475 | 3 | 0.81 |
| age | 0.5422 | 1 | 0.46 |
| sex | 0.0120 | 1 | 0.91 |
| LDL-C | 0.2279 | 1 | 0.63 |
| eGFR | 0.2993 | 1 | 0.58 |
| GGT | 0.0278 | 1 | 0.87 |
| NLR | 0.0107 | 1 | 0.92 |
| Uric acid | 0.2436 | 1 | 0.62 |
| Hypertension | 0.4045 | 1 | 0.52 |
| Diabetes | 0.2422 | 1 | 0.62 |
| GLOBAL | 3.2375 | 12 | 0.99 |

Schoenfeld residuals were used to test the proportional hazards assumption. A non-significant *P* value (*P* > 0.05) indicates no evidence of violation. df, degrees of freedom.

**Table S3**. Sensitivity analysis excluding longitudinal participants with less than one year of follow-up, unless an incident SCAS event occurred within this period.

| **Phenotype** | **HR (95% CI)** | ***P* value** |
| --- | --- | --- |
| MHNW | 1 (reference) | – |
| MHO | 1.14 (0.94–1.39) | 0.185 |
| MUO | 1.39 (1.11–1.73) | 0.004 |
| MUNW | 1.72 (1.14–2.59) | 0.010 |

Reference category: MHNW. Adjusted for age, sex, LDL-C, eGFR, GGT, NLR, and uric acid. Sample size after exclusion: n = 3,998 (original longitudinal subcohort, n = 4,938). HR, hazard ratio; CI, confidence interval; MHNW, metabolically healthy normal weight; MHO, metabolically healthy overweight/obesity; MUNW, metabolically unhealthy normal weight; MUO, metabolically unhealthy overweight/obesity.
**Table S4.** Age-stratified associations between metabolic-obesity phenotype and prevalent subclinical carotid atherosclerosis.

| **Age group** | **Phenotype** | **OR (95% CI)** | ***P* value** |
| --- | --- | --- | --- |
| <50 years | MHO | 1.38 (1.14–1.66) | <0.001 |
|  | MUO | 1.82 (1.48–2.25) | <0.001 |
|  | MUNW | 2.33 (1.39–3.76) | <0.001 |
| 50–60 years | MHO | 1.21 (1.07–1.37) | 0.002 |
|  | MUO | 1.80 (1.57–2.05) | <0.001 |
|  | MUNW | 2.06 (1.60–2.65) | <0.001 |
| ≥60 years | MHO | 1.09 (0.96–1.23) | 0.188 |
|  | MUO | 1.45 (1.26–1.67) | <0.001 |
|  | MUNW | 1.76 (1.39–2.26) | <0.001 |

Reference category: MHNW within each age stratum. Adjusted for sex, LDL-C, eGFR, GGT, NLR, and uric acid. Phenotype × age-group interaction *P* = 0.029. OR, odds ratio; CI, confidence interval; MHNW, metabolically healthy normal weight; MHO, metabolically healthy overweight/obesity; MUNW, metabolically unhealthy normal weight; MUO, metabolically unhealthy overweight/obesity.

**Table S5.** Follow-up duration and incident SCAS by metabolic-obesity phenotype (longitudinal subcohort, n = 4,938).

| **Phenotype** | **N** | **Incident SCAS, n (%)** | **Median follow-up, years (IQR)** |
| --- | --- | --- | --- |
| MHNW | 2,338 | 240 (10.3) | 1.47 (1.01–2.10) |
| MHO | 1,576 | 213 (13.5) | 1.61 (1.02–2.17) |
| MUNW | 124 | 26 (21.0) | 1.39 (1.04–2.04) |
| MUO | 900 | 154 (17.1) | 1.53 (1.01–2.11) |

Overall, the median follow-up duration was 1.78 years (IQR, 1.02–2.12), with 633 incident SCAS events among 4,938 participants (12.8%). Incidence values represent the proportion of participants who developed incident SCAS during follow-up (events/N × 100) and are not person-time incidence rates.

**Table S6.** Sensitivity analysis excluding covariates overlapping with metabolic phenotype definitions (reference category: MHNW).

**Cross-sectional**

| **Phenotype** | **Model** | **OR (95% CI)** | ***P* value** |
| --- | --- | --- | --- |
| MHO | Primary (with SBP) | 1.102 (1.015–1.196) | 0.0203 |
| MHO | Sensitivity (without SBP) | 1.170 (1.079–1.269) | <0.001 |
| MUNW | Primary (with SBP) | 1.545 (1.300–1.836) | <0.001 |
| MUNW | Sensitivity (without SBP) | 1.745 (1.471–2.070) | <0.001 |
| MUO | Primary (with SBP) | 1.356 (1.235–1.490) | <0.001 |
| MUO | Sensitivity (without SBP) | 1.574 (1.439–1.722) | <0.001 |

**Longitudinal**

| **Phenotype** | **Model** | **HR (95% CI)** | ***P* value** |
| --- | --- | --- | --- |
| MHO | Primary (with HTN+DM) | 1.138 (0.936–1.384) | 0.196 |
| MHO | Sensitivity (without HTN/DM) | 1.136 (0.935–1.380) | 0.199 |
| MUNW | Primary (with HTN+DM) | 1.692 (1.118–2.562) | 0.013 |
| MUNW | Sensitivity (without HTN/DM) | 1.730 (1.147–2.609) | 0.009 |
| MUO | Primary (with HTN+DM) | 1.355 (1.075–1.708) | 0.010 |
| MUO | Sensitivity (without HTN/DM) | 1.380 (1.107–1.722) | 0.004 |

Cross-sectional models included age, sex, LDL-C, eGFR, GGT, NLR, and uric acid in both the primary and sensitivity models; the primary model additionally included SBP, whereas the sensitivity model excluded SBP. Longitudinal models included age, sex, LDL-C, eGFR, GGT, NLR, and uric acid in both models; the primary model additionally included hypertension and diabetes, whereas the sensitivity model excluded these covariates. Point estimates for all three phenotypes were numerically higher in the sensitivity models.

**Figure S1.** Flow diagram of participant selection for the cross-sectional and longitudinal analyses.

**Figure S2.** Kaplan-Meier curves for cumulative incidence of subclinical carotid atherosclerosis according to metabolic-obesity phenotype.

Cumulative incidence of incident SCAS is shown for metabolically healthy normal weight (MHNW), metabolically healthy overweight/obesity (MHO), metabolically unhealthy normal weight (MUNW), and metabolically unhealthy overweight/obesity (MUO). Curves are truncated at 2 years because relatively few MUNW participants remained under observation beyond this time point. Differences among groups were assessed using the log-rank test (P < 0.001). Numbers at risk are shown below the x-axis. MHNW, metabolically healthy normal weight; MHO, metabolically healthy overweight/obesity; MUNW, metabolically unhealthy normal weight; MUO, metabolically unhealthy overweight/obesity.
